# Syndrome of Inappropriate Antidiuresis Due to Chronic Perforated Diverticulitis With Colovesical Fistula

**DOI:** 10.7759/cureus.35007

**Published:** 2023-02-15

**Authors:** Guillermo Ropero-Luis

**Affiliations:** 1 Department of Internal Medicine, Hospital de la Serranía, Ronda, ESP

**Keywords:** siadh, urinary bladder infiltration, chronic diverticulitis, fecaluria, syndrome of inappropriate antidiuresis, colovesical fistula, perforated diverticulitis, hyponatremia

## Abstract

A 54-year-old man presented with symptoms of dysuria and cloudy urine, as well as a history of passing stool in urine and repeated urinary tract infections over the past three months. He had previously undergone surgery for diverticulitis with secondary peritonitis 22 years prior. At the time of examination, the patient was in good general condition and euvolemic and had a plasma sodium level of 128 mmol/L and abnormally elevated urinary osmolality. Treatment involved oral urea and drinking according to thirst. After ruling out hypothyroidism and secondary adrenal insufficiency, the patient was diagnosed with syndrome of inappropriate antidiuresis (SIAD). A neoplastic-like mass in the sigmoid colon that infiltrated the bladder floor with a fistulous tract was found. The patient underwent a planned surgical intervention and maintained eunatremia without urea treatment postoperatively. The biopsy showed diverticulosis with intense inflammation in the submucosa but no neoplasia. This is the first reported case of SIAD associated with a colovesical fistula due to chronic perforated diverticulitis.

## Introduction

Syndrome of inappropriate antidiuresis (SIAD), traditionally known as syndrome of inappropriate secretion of antidiuretic hormone, is a prevalent yet frequently overlooked disorder. It is linked to multiple conditions such as neoplasms, infections, inflammatory processes, neurological diseases, and drugs [1]. This article presents the case of a patient with SIAD attributed to a previously unreported cause, to the best of the author's knowledge, a chronic perforated diverticulitis with a colovesical fistula.

## Case presentation

A 54-year-old man presented to the emergency department reporting dysuria and cloudy urine containing fecaloid materials. He was a regular smoker of about 30 cigarettes per day, drank alcohol occasionally, and had a history of surgery for acute diverticulitis with secondary peritonitis 22 years earlier; no other relevant history was mentioned. He was not taking any medications. He reported a three-month history of intermittent passing of stool in urine and repeated urinary tract infections (UTIs) that worsened prior to the visit. He denied symptoms such as dizziness, fatigue, lack of appetite, gait disturbance, or headache. He had been drinking three liters of water daily, per his family doctor's recommendation, to prevent the infections. A blood test six weeks prior to this episode showed plasma sodium (Na_P_) of 134 mmol/L (reference range: 135-145). Two weeks before this episode, he visited the emergency department for abdominal pain and low-grade fever, and a blood test showed Na_P_ of 126 mmol/L with normal glucose, urea, and creatinine at the lower limits of normal and no other significant alterations. No further investigation of hyponatremia was performed, and no specific treatment was prescribed, only empiric antibiotherapy with ciprofloxacin.

On examination, the patient was in good general condition, normotensive, and afebrile, with no signs of hypovolemia or hypervolemia. Rectal exam showed induration of the anterior wall. Lab tests showed Na_P_ 128 mmol/L, urea 30 mg/dL (reference range 20-50), creatinine 0.8 mg/dL (reference range 0.7-1.3), glucose 99 mg/dL (reference range 70-110), measured plasma osmolality (Osm_P_) 270 mOsm/kg (reference range 280-295), urinary sodium (Na_U_) 31 mmol/L, and urinary osmolality (Osm_U_) 195 mOsm/kg.

The patient was admitted with moderate euvolemic hyponatremia consistent with SIAD, fecaluria possibly from rectovesical fistula, and complicated UTIs. Treatment for hyponatremia involved oral urea (15 g/12 h) and drinking as per thirst. Ceftriaxone was initiated as empirical antibiotherapy and changed to amoxicillin-clavulanic acid after extended-spectrum beta-lactamase-producing *Escherichia coli* was detected in urine culture.

**Figure 1:**
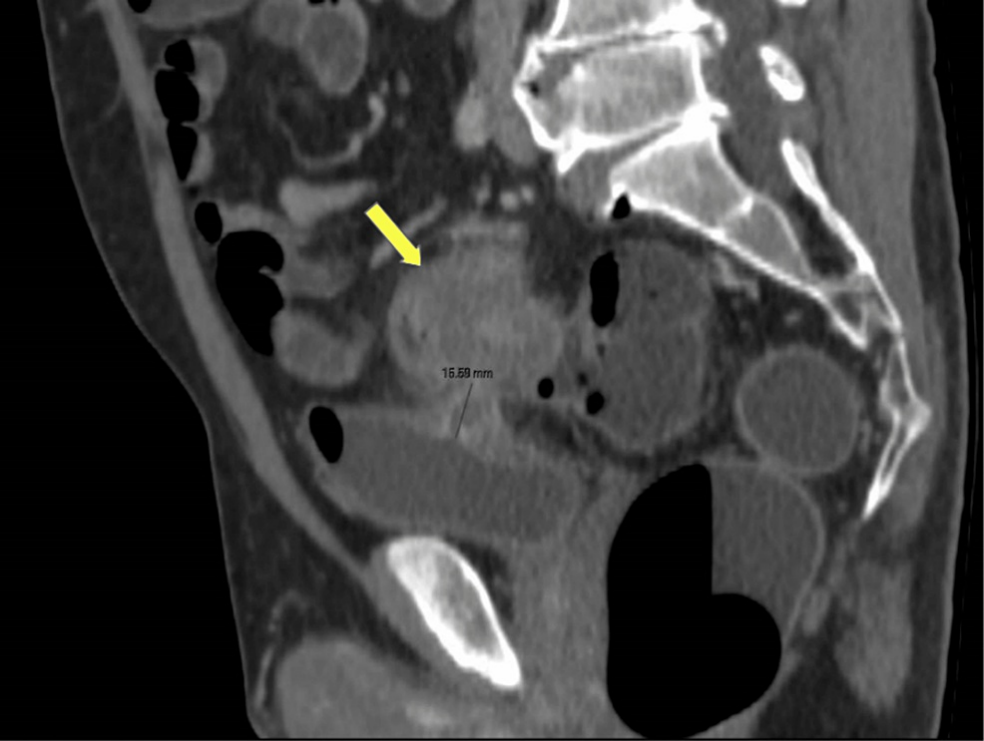
Sagittal view of the abdominal CT scan showing the mass in the sigmoid colon (arrow) and the colovesical fistula (annotation). CT: Computed tomography.

**Figure 2:**
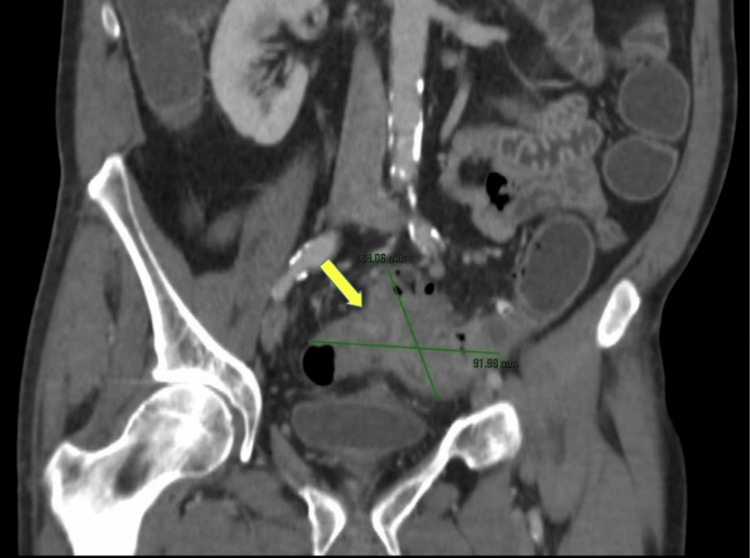
Coronal section of the abdominal CT scan showing the dimensions (annotations) of the mass in the sigmoid colon (arrow). CT: Computed tomography.

The diagnosis of SIAD was confirmed after ruling out hypothyroidism (thyrotropin 1.3 mIU/L, reference 0.4-5) and secondary adrenal insufficiency (basal cortisol 25 mcg/dL, reference 5-25). Evaluation for rectal/bladder neoplasia was conducted. A chest-abdomen computed tomography scan and virtual colonoscopy showed a neoplastic-like mass in the sigmoid colon, about 26 cm from the anal margin, that infiltrated the bladder floor with a fistulous tract (Figures 1, 2), severe diverticulosis, and suspicious locoregional adenopathies. However, sigmoidoscopy showed a normal mucosa, extensive diverticulosis, and significant adhesive syndrome, suggesting diverticular or extracolonic origin of the mass. Flexible cystoscopy revealed a normal bladder except for the fistulous orifice.

Regarding the evolution of hyponatremia, it was initially corrected with urea treatment, reaching Na_P_ 136 mmol/L on the third day of treatment. However, after undergoing preparation for colonoscopy, it decreased to 129 mmol/L, rising two days later to 137 mmol/L. The use of tolvaptan may have prevented or minimized the relapse of hyponatremia suffered during colonoscopy preparation due to high fluid intake, but it was not available at our center at that time.

The biopsies taken during the sigmoidoscopy showed fragments of the large intestine with focal intense inflammation in the submucosa, without evidence of neoplasia. The case was discussed in the multidisciplinary digestive tumors committee, and a planned surgical intervention was decided upon. The patient was discharged with Na_P_ 138 mmol/L, on treatment with urea 15 g/12 h and instructed to drink according to thirst.

**Figure 3:**
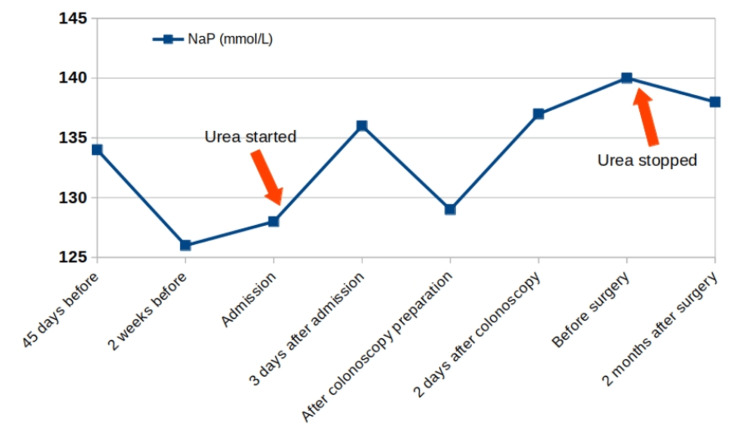
Trend in plasma sodium levels throughout the process. NaP: Plasma sodium.

The surgery was performed nine days after discharge. The preoperative Na_P_ was 140 mmol/L. An infra-umbilical median laparotomy was performed, resulting in an oncological sigmoidectomy, terminal-terminal colorectal anastomosis, and resection of the fistulous tract with repair of the bladder wall, without complications. During the postoperative period, the patient maintained normal natremia without urea treatment. The pathologic analysis of the surgical specimen showed a segment of the large intestine with intense acute exacerbation of chronic inflammation and presence of diverticula perforated by diverticulitis, without evidence of neoplasia. A follow-up blood test performed two months later showed Na_P_ of 138 mmol/L. The evolution of Na_P_ throughout the process can be seen in Figure 3.

## Discussion

This is the first case described of SIAD associated with a colovesical fistula due to chronic perforated diverticulitis. Several criteria are required for the diagnosis of SIAD [1], all of which were met by the patient: Na_P_ less than 135 mmol/L with effective Osm_P_ less than 275 mOsm/kg, Osm_U_ greater than 100 mOsm/kg with preserved renal function, Na_U_ greater than 30 mmol/L in conditions of sufficient sodium intake, clinical euvolemia, and exclusion of other causes: use of diuretics, hypocortisolism, hypothyroidism, and non-osmotic physiological stimuli of antidiuretic hormone (ADH) secretion such as nausea and pain.

After an exhaustive literature search in different databases, we only found one retrospective observational study, which analyzed patients over 50 years old with acute diverticulitis and appendicitis, where a higher prevalence of hyponatremia was found in patients with intestinal perforation (29% compared to 16%) [2]. However, their characteristics are not described, so it is impossible to know how many could correspond to SIAD, and if they had other factors that could be contributing to its development.

In the case of our patient, other etiologies (tumoral, pharmacological, etc.) were ruled out, so the development of SIAD was attributed to the colonic inflammatory process and the colovesical fistula. This suspicion was confirmed after the surgical intervention that resolved the SIAD. The underlying pathophysiological mechanism in this case is not clear, although it is possible that it is similar to other inflammatory processes that stimulate the non-osmotic secretion of ADH. SIAD is also described in the context of acute urinary retention, probably due to stimulation of ADH secretion by bladder distension [3,4]. It is possible that in our case, infiltration of the bladder wall by the inflammatory mass stimulated ADH by a similar mechanism.

## Conclusions

In conclusion, the case presented is the first reported case of SIAD associated with a colovesical fistula due to perforated chronic diverticulitis. The patient met all criteria for the diagnosis of SIAD, and other causes were ruled out. The development of SIAD was attributed to the colonic inflammatory process and the colovesical fistula. Although the exact mechanism is unclear, it is possible that it is similar to other inflammatory processes and bladder distension that stimulate non-osmotic ADH secretion. The resolution of the SIAD after surgical intervention confirms this suspicion. Further studies are needed to shed light on the pathophysiology of this case and the potential relationship between colonic inflammatory processes and SIAD.
